# Uncovering the hidden implications of water scarcity for maternal health: a photovoice study in rural Malawi (Thyolo district)

**DOI:** 10.3389/fgwh.2025.1588219

**Published:** 2025-06-09

**Authors:** Chiara Pittalis, Elizabeth Kogoya, Antonio Jaén Osuna, Christabel Kambala

**Affiliations:** ^1^ Institute of Global Surgery, School of Population Health, RCSI University of Medicine and Health Sciences, Dublin, Ireland; ^2^ Public and Environmental Health Sciences Department, School of Science and Technology, Malawi University of Business and Applied Sciences, Blantyre, Malawi

**Keywords:** clean water, public health, climate change, Africa, community-based participatory research, photovoice

## Abstract

**Introduction:**

Access to clean water is essential for health, but in rural Malawi, water scarcity significantly threatens the health of postpartum women and their babies. Water is critical not only for hydration but also for hygiene, preventing infections, and promoting overall health, particularly during the postpartum period. Despite women's central role in water management within their households, they are seldom included in discussions about water access and management at the community level or within formal governance structures, where decisions are often made without their input. As climate change exacerbates water scarcity, addressing these challenges becomes increasingly urgent for maternal and child health.

**Goal & methods:**

This study aimed to create space for postpartum women to document and share their experiences with water scarcity and its implications through participatory photography. Eight women from rural communities in Thyolo District, Southern Malawi, were trained in photography and ethics. They captured images reflecting the challenges they faced with water access and maternal health. Through gallery walks and group discussions, photovoice allowed women to share the stories behind their images, providing deeper insight into the emotional, social and health implications of water insecurity. A phenomenological analysis was conducted to interpret the photographs and narratives.

**Results:**

The women's photographs and stories highlighted the multifaceted impact of water scarcity on health. They depicted not only the physical toll of water collection but also the emotional stress and heightened risk of infection due to inadequate sanitation. Through photovoice, the women conveyed a powerful narrative of vulnerability, resilience and frustration, revealing issues that are often invisible in traditional health research.

**Conclusion:**

Photovoice proved to be an effective tool for amplifying the voices of marginalized postpartum women, enabling them to highlight the urgent need for clean water access. This approach not only raised awareness of water-related health issues but also provided valuable insights into the lived realities of women in rural Malawi. As climate change continues to intensify water scarcity, these findings underscore the need for sustainable, community-driven solutions. The study offers a model for inclusive research on global health challenges, particularly in vulnerable communities.

## 1 Introduction

The Climate Crisis makes access to water scarce and unpredictable, especially in rural sub-Saharan Africa (SSA) where the most vulnerable populations live. Annually 17 million women in SSA give birth in healthcare facilities (HCFs) without adequate water to provide safe care, and low-income countries such as Malawi are most affected (1).

Despite the increase in access to protected water sources (e.g., boreholes) in rural areas of Malawi—from 64% in 2005–88.3% in 2020—distribution, maintenance and functionality of water points remain an issue, and 11.7% of the population still relies primarily on unprotected water sources (2). Water quality is also under threat due to increasing levels of sedimentation, biological and chemical contamination, effluent discharges and solid waste contamination (2). The frequency of extreme weather events in Malawi poses further risks for its water sources (3). This precarious access to potable water has serious implications for maternal and neonatal health outcomes (2, 4–6).

To mitigate the situation, a new initiative, SURG-Water, is testing an innovative technology to improve access to clean water for maternal care in Malawi, which involves solar disinfection of rainwater (7). While SURG-Water aims to address the immediate water needs of expectant mothers receiving care at rural health facilities, it is also important to understand women's experiences in the postpartum period, once back to their communities, to identify additional needs that may require attention. Clean water is indispensable for mothers due to its role in preventing infections, supporting breastfeeding (8), and ensuring the health and safety of newborns (4, 9). For most mothers, the postpartum period is particularly demanding due to biological, physical, social and emotional changes, and can be even more so when there is a lack of clean running water to meet their necessities and those of their families (10, 11).

However, very little is known about these challenges at present. Studies in SSA often are limited to measuring access to safe drinking water (12, 13) or examining mothers' knowledge and practices regarding safe drinking water (14–16), mostly based on quantitative methods. There is a paucity of studies on wider aspects of water utilization and their implications, and on the perspectives of postpartum women and their families (11). Despite the centrality of women in water management within their households, they are rarely given the opportunity to express their views or participate in discussions concerning water management in their communities or within formal governance structures, where decisions are often made without their input (17). Their health is impacted by water-related issues, yet this is still an under-researched area (17).

In this backdrop, the aim of this study was to obtain a thorough understanding of how women in the postpartum period meet their needs for water, examining the interplay of factors affecting water acquisition and utilization. This will not only inform our project but can also support other initiatives to tackle water insecurity in Malawi. The focus was on bringing to light the lived experience of mothers in rural communities, through a photovoice approach (18).

The use of photovoice in research concerning health and water, sanitation, hygiene (WASH) in Sub-Saharan Africa has gained popularity as a valuable method to capture the complexity of water issues (19). Studies across the region have found photovoice useful in providing insights into social norms within rural communities (20), highlighting the gendered nature of water (21) and giving a voice to the most marginalized community members (18).

## 2 Methods

This study was undertaken in the period February to May 2024, as part of the larger SURG-Water project.

### 2.1 Research context

The research took place in Thyolo district communities served by Chimvu Health Centre and Thekerani Rural Hospital, where the SURG-Water project operates. Thyolo, in Southern Malawi, is vulnerable to floods, droughts and cyclones due to its location near cyclone paths, hilly terrain and environmental degradation (22, 23). Floods often damage roads and bridges, isolating communities (24, 25). As one of Malawi's poorer districts (26), many households depend on subsistence farming and have limited resources for recovery. The growing population [increase of over 30% in the country from 2013–2023 (27)] adds pressure on water resources, further increasing vulnerability. The research sites were selected through consultations with the District Health Management Team and a situation assessment that identified them as facing particularly severe water access challenges. While they may not represent the full breadth of the region or the national context, they provide insights into the water-related issues in a highly vulnerable area.

### 2.2 Approach

The photovoice method was chosen for its effectiveness in participatory needs assessments (18), allowing participants to use cameras to visually document issues that affect them, which supports reflection and dialogue across cultural and language barriers (18, 28)—ideal for low-literacy settings like rural Malawi. Rooted in community-based participatory action research, photovoice fosters collaboration, empowerment and equal partnership (29) with community members, who actively contribute to the research process. In doing so, the focus is shifted from researchers to those with lived experience (30). Another advantage is that this approach not only identifies community needs but also highlights assets, enabling the integration of gained knowledge into actions that benefit the community (18).

Implementing photovoice, however, can be challenging due to the need to build meaningful partnerships, address power imbalances and encourage participation (18, 31–33). This is particularly relevant in rural settings, like Thyolo, where low literacy [one third of the population (34)], modest living conditions, and strong social norms may create hesitancy toward engaging with “outsiders”. Our study deliberately tried to mitigate these challenges throughout the photovoice process (35, 36)—as described below.

#### 2.2.1 Preparations

The first step focused on reaching consensus with the communities on the research plan. Building a positive, balanced partnership from the start involved addressing power differences between academic and community partners. We tried to do so in two ways.

Firstly, international (CP) and local (CK, EK) female researchers were equally involved in the study, each bringing unique expertise. CP, an experienced senior researcher with a PhD in health research, is trained in participatory and anticolonial methods, and has over seven years of experience in health research in Sub-Saharan Africa. CK, a senior Malawian academic with a PhD in public health, specializes in environmental and maternal health research, including photovoice, while EK, a Malawian research assistant currently completing her master's degree, brings expertise in water issues. Together, their collective expertise created a well-rounded team for this study.

Secondly, community partners were involved from the outset. SURG-Water, using a participatory approach, facilitated connections with Chimvu and Thekerani communities in early 2023, establishing an ongoing relationship. Selection of women co-researchers was done jointly, in consultation with the local health centers to identify suitable candidates, based on three criteria: (1) being mothers or relatives/guardians of mothers who had recently given birth at the Chimvu or Thekerani health facilities and with first-hand experience of water issues; (2) being available to commit to participate in the study for its full duration; (3) being willing to share their experiences with an outside audience.

Eight women were selected as co-researchers for the photovoice study—four mothers and four guardians. Only one of the mothers was a first-time parent, while the others had multiple children. Their ages ranged from 20–43 years, and they represented various villages served by the Chimvu and Thekerani health facilities.

Community collaborators and co-researchers contributed to shaping the direction of the study and provided input during workshops at each site, leading to adjustments in the research plan. For instance, due to severe water scarcity, mothers had to balance water use between childcare and other essential household chores. Co-researchers expressed a desire to document this careful balancing act, so it was agreed that the photos could depict both maternal and other daily activities.

All workshops were conducted in Chichewa, a local language widely spoken in Malawi, with translation into English by the local research assistant as necessary. This was to maximize participation of community co-researchers, to ensure they fully understood the photovoice concept and aims of the study, and could confidently ask questions or input.

We kept our research approach flexible to better meet the co-researchers' needs and navigate social dynamics. For instance, local leaders (two in Chimvu, one in Thekerani) joined the workshops, though this was initially unplanned to reduce potential bias. The co-researchers, unfamiliar with research and hesitant to speak in new settings, benefited from this support. In rural areas, it is unusual for women to attend such gatherings alone, as it may be frowned upon by husbands and authority figures. The leaders encouraged the women's active participation, emphasizing the value of voicing their needs to foster community change.

#### 2.2.2 Taking the photos

Co-researchers were trained on using disposable cameras, the types of images to capture, and the ethics of photography, with the support of a well-experienced communication specialist (AJO, male). Each received a camera with a capacity for 37 photos and given 5 days to take the photos. After completing the task, they shared their experiences. While most worked independently, the Chimvu co-researchers initially consulted each other to clarify camera use. In general, they did not report encountering any major difficulties in taking the photographs. Once the project's purpose was explained, most people agreed to be photographed. One co-researcher focused on her own village, while others visited nearby areas to capture shared challenges. Two Thekerani co-researchers photographed multiple locations to find enough willing photo subjects.

#### 2.2.3 Joint discussion and analysis of photographic data

Given the study's socio-cultural context, a group approach to critical reflection, rather than an individual one, was used to encourage the co-researchers to share their views in a safe, familiar setting. This group work was a guided critical thinking process, i.e., a stepwise process to ease the co-researchers into talking and reflecting about the material (35).

In a series of “gallery walks”, during dedicated half-day workshops, each co-researcher selected the photographs they considered most significant from all the rolls, reporting back to the group the reason for their choices after each round. Once the co-researchers were satisfied with the selection, they engaged in a group dialogue session to discuss the meaning of their images and to contextualize them. The PHOTO questions (37) broadly guided the discussion: describe your Picture; what is Happening; why this phOto; what it Tells about your life; and Opportunities for improvement.

While the questions were directed to the author of each photo, co-researchers could contribute to the discussion, and each photo received a title to summarize its main message (or one title for multiple photos when these were very similar) and a brief narrative. Recurring themes or concepts were collectively identified. Themes related to water access, physical demands, and emotional strain emerged consistently across the co-researchers' contributions. This repetition suggested that saturation had been reached, as the key themes were repeatedly articulated in both the visuals and narratives. Out of the 140 photos taken by the Chimvu group and 141 by Thekerani, co-researchers ultimately selected 21 and 23 images, respectively.

A phenomenological analysis (38) of the data was done. NVivo 12 software was used to organize the data and assist in identifying emerging patterns. However, the analysis remained rooted in phenomenological interpretation, with NVivo serving to facilitate rather than drive the process. Coding was done by the academic researchers, integrating overarching themes and key issues (from photo titles) identified by the co-researchers with additional codes based on “bottom up” patterns observed in the photo narratives and group discussions. This approach helped reveal additional layers of meaning related to shared challenges and values. Consensus on the themes was built through ongoing team discussions, and no major conflicting views emerged. Potential bias was mitigated by ensuring that the co-researchers' input guided the analysis, alongside validation with the community (see below).

#### 2.2.4 Validation with the communities

At the end of this process, the agreed final selection of photographs was exhibited at dedicated community meetings in Chimvu and Thekerani to share the outcomes of these efforts and give an opportunity to other community members to review and validate the material, and to reflect on whether there was anything missing. In Thekerani, in particular, some of the photo subjects had explicitly asked to see the final product. Hence, this exhibition was part of the steps taken to maintain a positive relationship and trust with local communities.

### 2.3 Ethical considerations

Ethical approval was granted by the Malawi National Committee on Research in the Social Sciences and Humanities. All co-researchers provided written informed consent to participate in this study and for the use of their photographs for research and dissemination purposes. Training exercises reinforced the need to obtain informed consent from subjects prior to taking photos and respect of ethical best practices. Co-researchers themselves made the decision about which photographs and captions could be used. They were not directly compensated for their work. However, refreshments were provided during the workshops and travel expenses reimbursed, a bag with some hygiene items was distributed as a thank-you gift and co-researchers were given printouts of their photos.

The use of photographic data can raise ethical concerns relating to the visual exposure of people, places and their privacy (36, 39), particularly when involving children. In this paper we have tried to select photos that do not directly show the faces of community members when possible, and do not contain sensitive or potentially contentious images. We have attributed all photographs and quotes to co-researchers using numerical codes to respect their privacy.

## 3 Results

The sample of 44 photos selected by the co-researchers captures salient moments of the typical life in the local villages. Within it, two interlinked types of images can be distinguished: those documenting the different sources of water available in the locality and water acquisition practices, and those showing how the water is used in the everyday activities of the women and their communities.

As in Table 1 below, multiple themes and key messages were identified by the co-researchers. Since there is large overlap across the two groups, for presentation purposes the material has been rearranged to create one coherent narrative and organized into two overarching concepts: water acquisition and water utilization.

**Table 1:** Themes and photo titles/key messages identified by co-researchers.

Chimvu group	*n* = 21 total photos	Thekerani group	*n* = 23 total photos
		**Fetching water from boreholes**	**5**
		Waiting/Kuyembekezera	
		Water difficulties/Mavuto a madz (2 photos)	
		Distance/Mtunda	
		Buckets/Zidebe	
		**Fetching water from unprotected sources**	**2**
		Unprotected well/Madzi a muchitsime chosatetezedwa	
		The well/Muchitsime	
**Baby and child hygiene**	**3**	**Baby bathing**	**2**
Minimal water/Madzi ochepa		Helping/Kuthandiza	
Boy child/Mwana wamamuna		Baby bathing/Kusambitsa mwana	
Man contribution—Abambo kutengapo mbali pa pa ukhondo wa mwana			
**Body hygiene and clothes**	**7**	**Washing clothes**	**2**
Seeking for water/Kufufuza madzi (2 photos)		Restaurant/Malo odyera	
We want clean water/Tikufuna madzi abwino		Nappies/Matewera	
Long distance and fights/Ulendo wautali ndi ndewu			
Body hygiene/Ukhondo wapathupi			
Struggle for water/Mavuto a madzi			
Water scarcity/Kusowa kwa madzi			
**Cooking**	**2**		
Cooking/Kuphika			
Water for cooking/Madzi ophikila			
**Household and utensils**	**5**	**Washing dishes**	**3**
Cholera prevention/Kupewa kolera		Hygiene/Ukhondo	
Disease prevention/Kupewa matenda		Washing dishes/Kutsuka ziwiya	
Nearby water source/Madzi opezeka pafupi		Gender/Jenda	
Utensils hygiene/Ukhondo wa ziwiya			
Household hygiene/Ukhondo wa pakhomo			
**Water for crops**	**2**	**Crops**	**2**
Source of income/Njila yopezela ndalama (2 photos)		Irrigation/Kuthirira (2 photos)	
**Drinking**	**2**	**Water for drinking**	**2**
Water is life/Madzi ndi moyo		Drinking water/Madzi okumwa (2 photos)	
Milk production/Kupanda mkaka			
		**Water for animals**	**2**
		Pigeons/Nkhunda	
		Cows/Ng'ombe	
		**Fish farming**	**2**
		Fish farming/Ulimi wa nsomba (2 photos)	
		**Washing bikes**	**1**
		Washing bikes/ Kutsuka njinga	

### 3.1 Water acquisition

The co-researchers (with the exception of one) reported that there is no water source within their villages. The photographic evidence captured by them documents their struggle in accessing clean and safe water, which is essential for many chores. Their daily routine often includes walking long distances and enduring physical strain to fetch water which is carried in large, heavy buckets (usually 20 liters) on their heads. The quality and safety of water are major concerns, as many families rely on either boreholes (protected sources) or unprotected sources, each presenting distinct challenges.

#### 3.1.1 Boreholes vs. unprotected water sources

**Figure 1:**
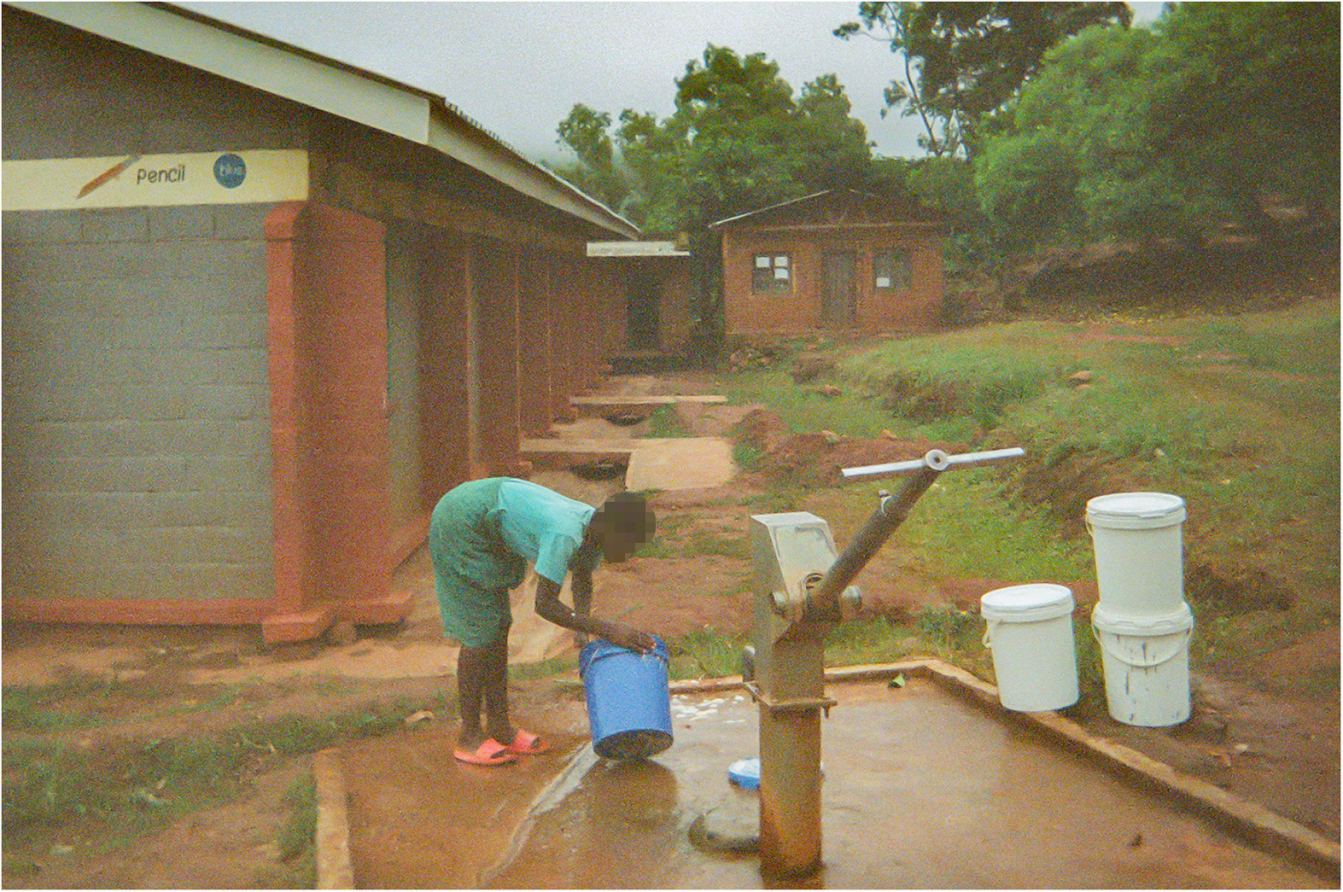
Buckets/Zidebe. This photo portrays a girl fetching water at the borehole. I wanted to capture the buckets to show that this is a very heavy task. The girl is preparing her buckets in advance while she waits for another girl to come and help her. She is doing this in advance to beat the queue, so when the others come she will already have several buckets filled to bring home. (Co-researcher 7).

Boreholes, which are deep wells with protective casings, offer a more reliable and generally safer water source by protecting water from surface contamination. As explained by the co-researchers, borehole water is considered by local communities critical for their health, as waterborne diseases, like Cholera, are prevalent in these areas and have taken many lives. However, boreholes are not available in every village, forcing women to travel farther. High demand leads to significant queues at boreholes increasing waiting times (see Figure 1), particularly during the dry season when water supply diminishes. Co-researchers reported that in shallow boreholes the water level goes down and it becomes more difficult to draw water. When boreholes break down, communities may lack the resources or expertise to fix them promptly, obliging women to revert to unprotected sources.

**Figure 2:**
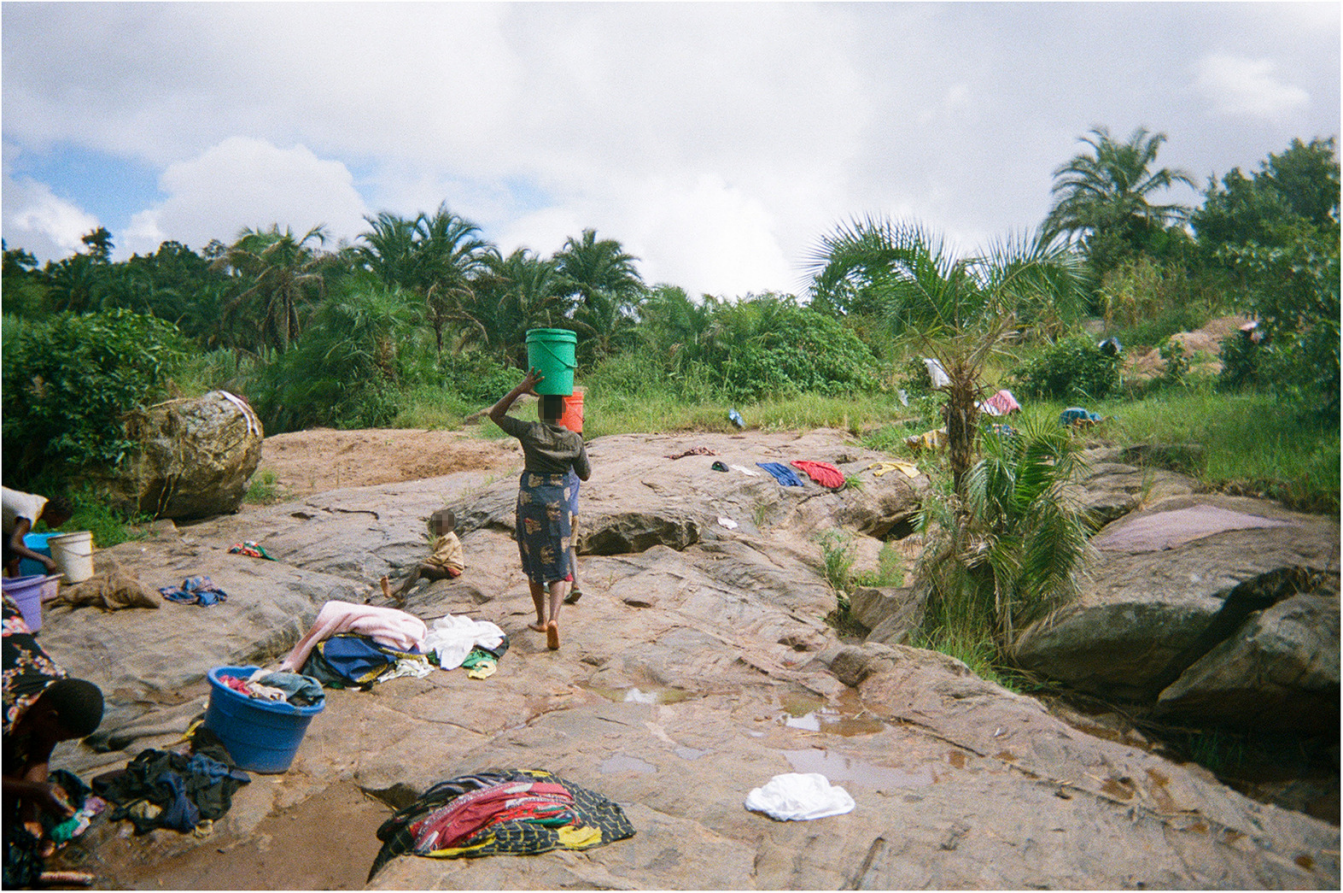
Struggle for water/Mavuto a madzi. This photo portrays women going to a river to wash their clothes. The women have to cross the river because the safe and accessible point to get to the water is on the other side. When there is a lot of rain, the river swells up so they cannot cross. This means they are unable to access the water. The problem is particularly common during the rainy season. There are no alternative solutions for the women, they have to wait until the river goes down again and be patient. (Co-researcher 3).

Unprotected sources, such as rivers, streams and makeshift wells, are generally available in most rural areas and do not require long waiting times or maintenance, providing a seemingly convenient alternative water source. As such, they are crucial to daily life but are deeply vulnerable to climate pattern shifts that create new risks for water accessibility and quality, especially for women and children who rely on them (see Figure 2). The quality of water worsens during droughts (when stagnant) or heavy rainfalls (which introduce contaminants into water sources), posing serious health risks from waterborne illnesses. As gathered from the co-researchers' narratives, local communities are very aware of these risks and occasionally people disinfect the water with chlorine tablets when possible, but often they simply have no option but to use the contaminated water.

#### 3.1.2 Water collection practices

**Figure 3:**
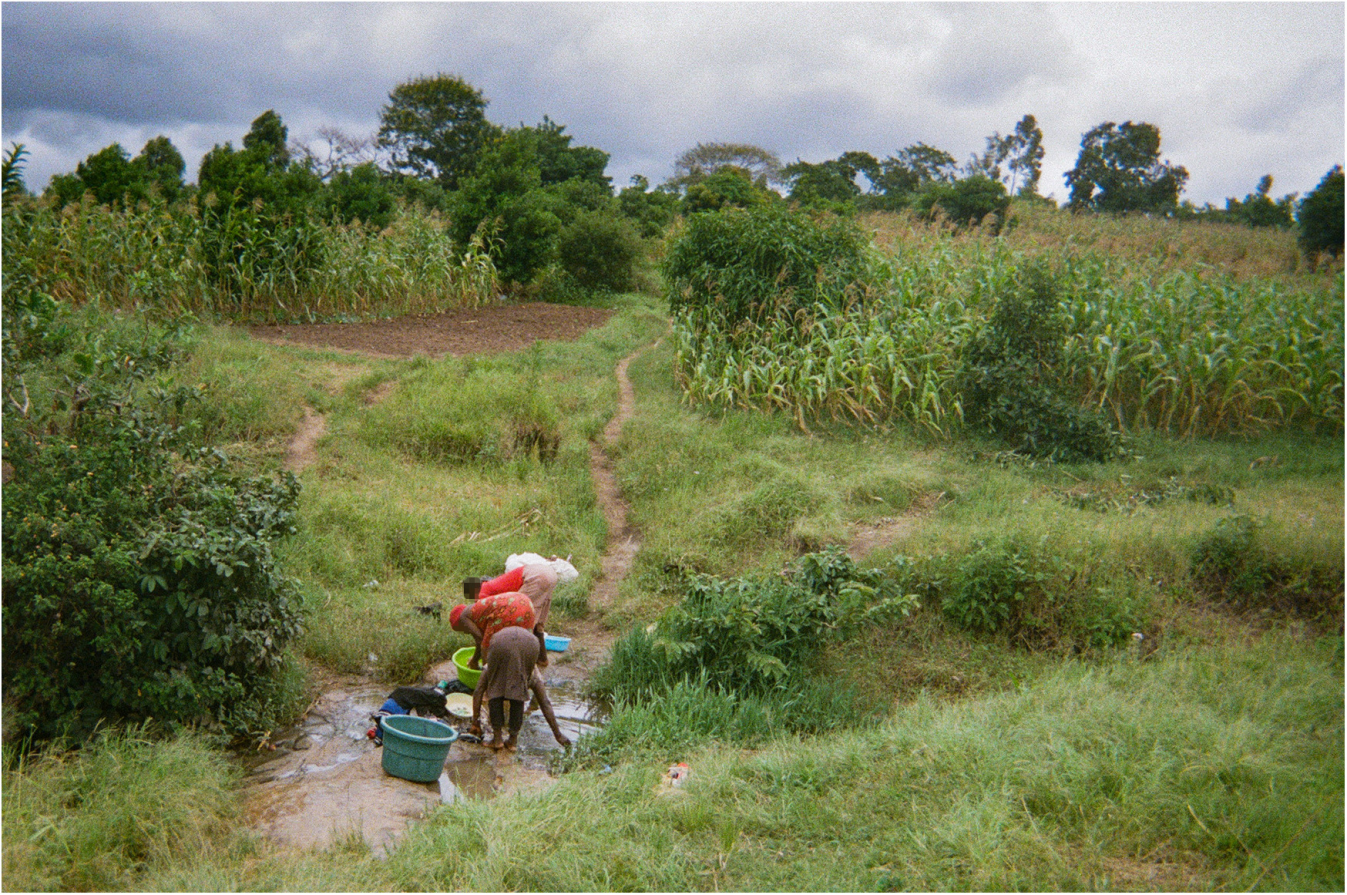
Long distance and fights/Ulendo wautali, ndewu. This photo shows a group of women fetching water from a small stream running through a field. I wanted to show how much people are struggling. It is always the responsibility of women and girls to fetch water. They have to walk long distances to reach this stream, and the water here is not clean so there is a risk of diseases. Sometimes women leave their homes at 2 am to get there and stay the whole day at the stream washing things. During the dry season months, especially in September, women have to go even further away to find water. This long time outside the home is often a cause of domestic fights, because the husbands ask “what took you so long?”. This happens a lot. (Co-researcher 4).

As explained by the co-researchers, in rural Malawi the burden of collecting water primarily falls on women. The difficulties of accessing water described above oblige women to leave their families unattended for long times, taking away from other responsibilities. This can disrupt family dynamics and exacerbates stress within their households (see Figure 3).

**Figure 4:**
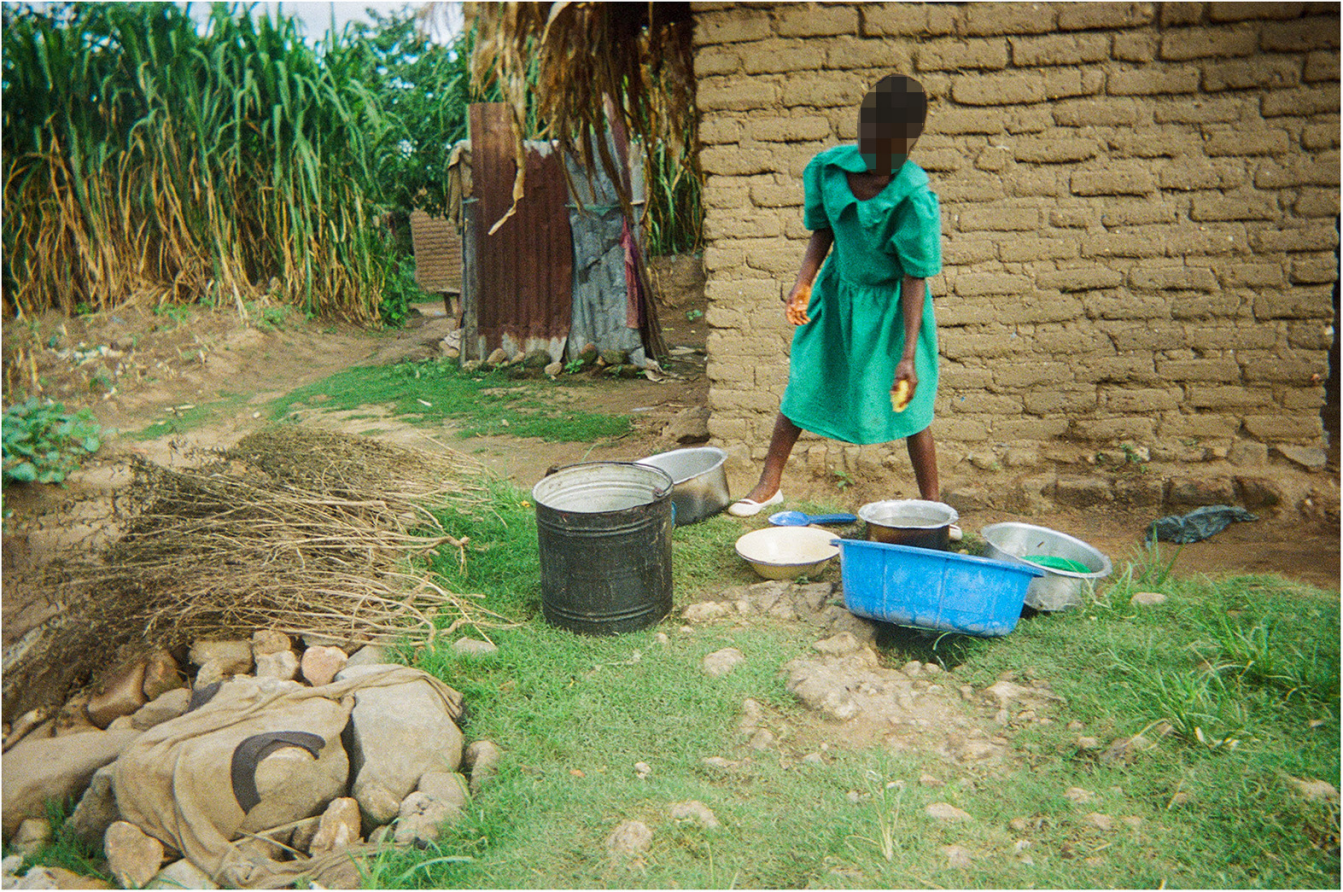
Nearby water source/Madzi opezeka pafupi. This photo portrays a girl who took water from a nearby river to wash dishes. The girl knows the importance of getting water from clean water sources but this is not always possible. So the photo shows this struggle. The girl is still wearing her uniform because she is just back from school. She will clean the dishes after school before going to play. This is normal, it is her chore and contribution to the family. If she doesn't do it, she will not be allowed to eat her dinner that night. Everyone has to contribute to the family. (Co-researcher 2).

**Figure 5:**
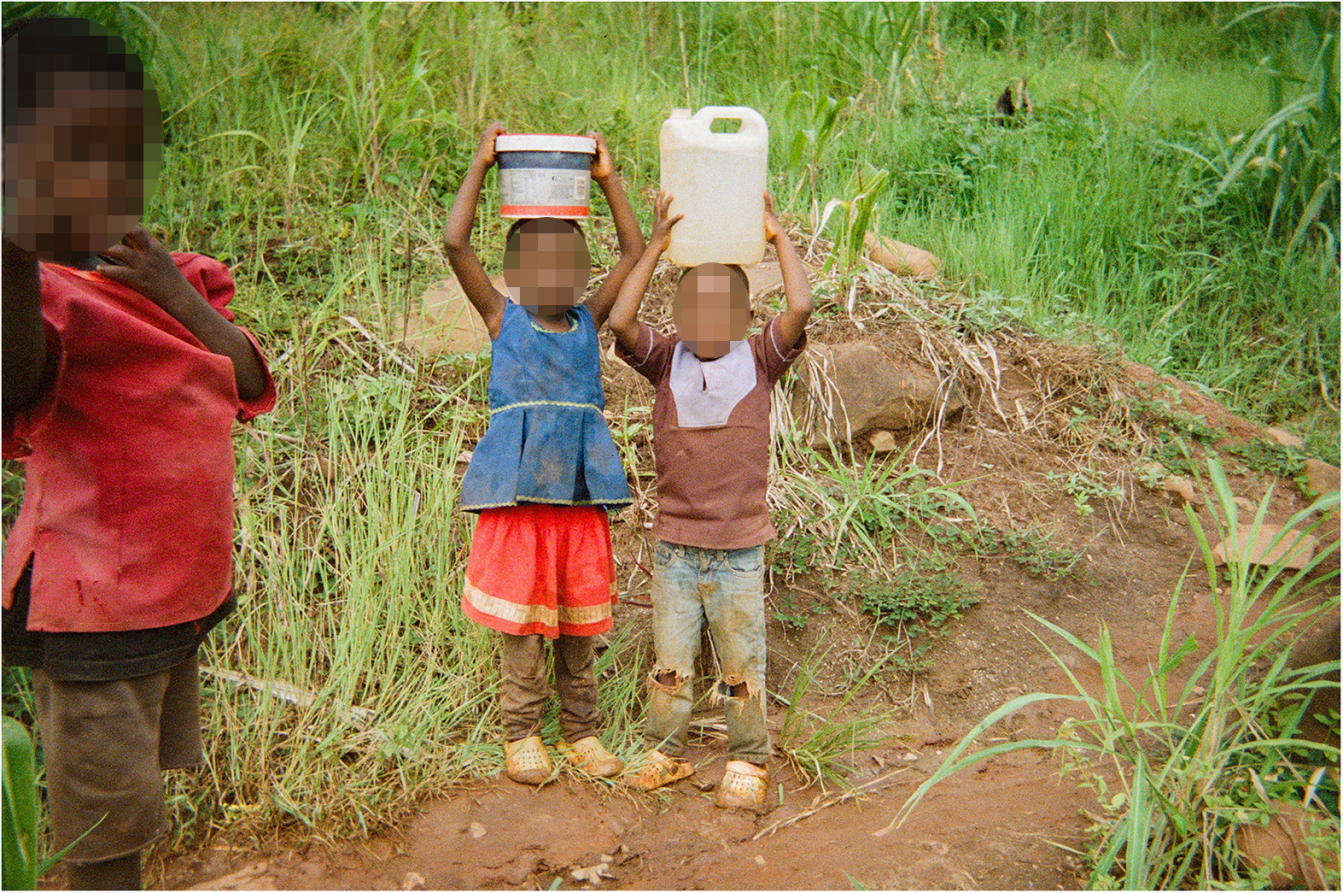
Distance/Mtunda. In this photo two kids are carrying water in containers on their heads. (…) The kids took the water far from home and had a long distance to walk back. In the local community, it is common for kids to fetch water for their family because the parents are often busy with farming. One of the kids (the boy in the brown shirt) is about to cry because he is struggling with the chore, nevertheless is aware of the importance of doing this task for his family. (Co-researcher 7).

Some help is provided by children (Figures 4,5), who play a significant role in supporting their families, contributing to household duties from a young age. In the photographs there was evidence of children helping with water collection, as well as bathing younger siblings, washing the dishes after meals and cleaning the house. This sense of family contribution is a core part of their upbringing and reflects strong values of cooperation, responsibility and resilience evident from the narratives of the co-researchers. Once boys grow up and get married, however, their chores change and they no longer help with fetching water.

### 3.2 Water utilization

Water is critical for the overall well-being of mothers and their infants, but its limited availability forces families to make challenging decisions about how to allocate this resource. As described by co-researcher 6: “*in general, people in the village try to differentiate clean from unclean water when they can, and use each type for different tasks. The bucket with a cover contains clean borehole water and this is used for cooking and drinking. Often the unclean water is kept in the larger and uncovered buckets. This is used for other tasks. However, not everyone follows these rules*”. The issue is not just about how water of varying quality is used for different purposes, but also about the consequences of not having enough water in general. Co-researchers pointed out that the borehole water, in particular, is to be used sparingly given its high value (cleaner but difficult to collect).

**Figure 6:**
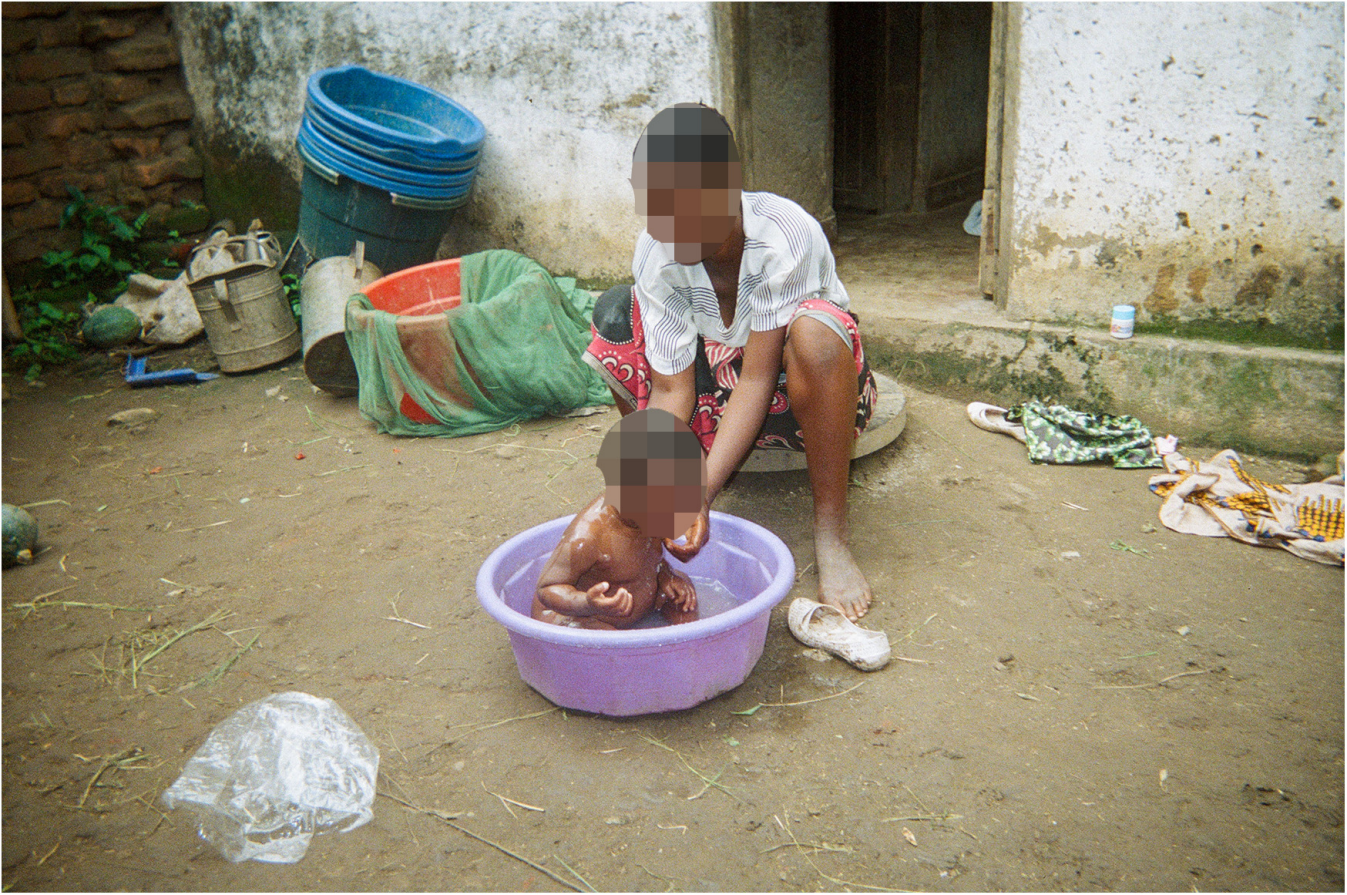
Minimal water/Madzi ochepa. The photo portrays a mother bathing her child. The empty buckets at the back show she does not have enough water. Even the little water in the bucket she is using is not much, but she is trying to do the best she can to look after her child. This water is from the river, so it is not clean. (Co-researcher 2).

For example, according to the co-researchers, bathing is one of the most important uses of water in the care of babies and ideally should be done using borehole water. Yet, the lack of clean, sufficient water means that compromises have to be made, which affect their ability to ensure proper baby hygiene (see Figure 6).

**Figure 7:**
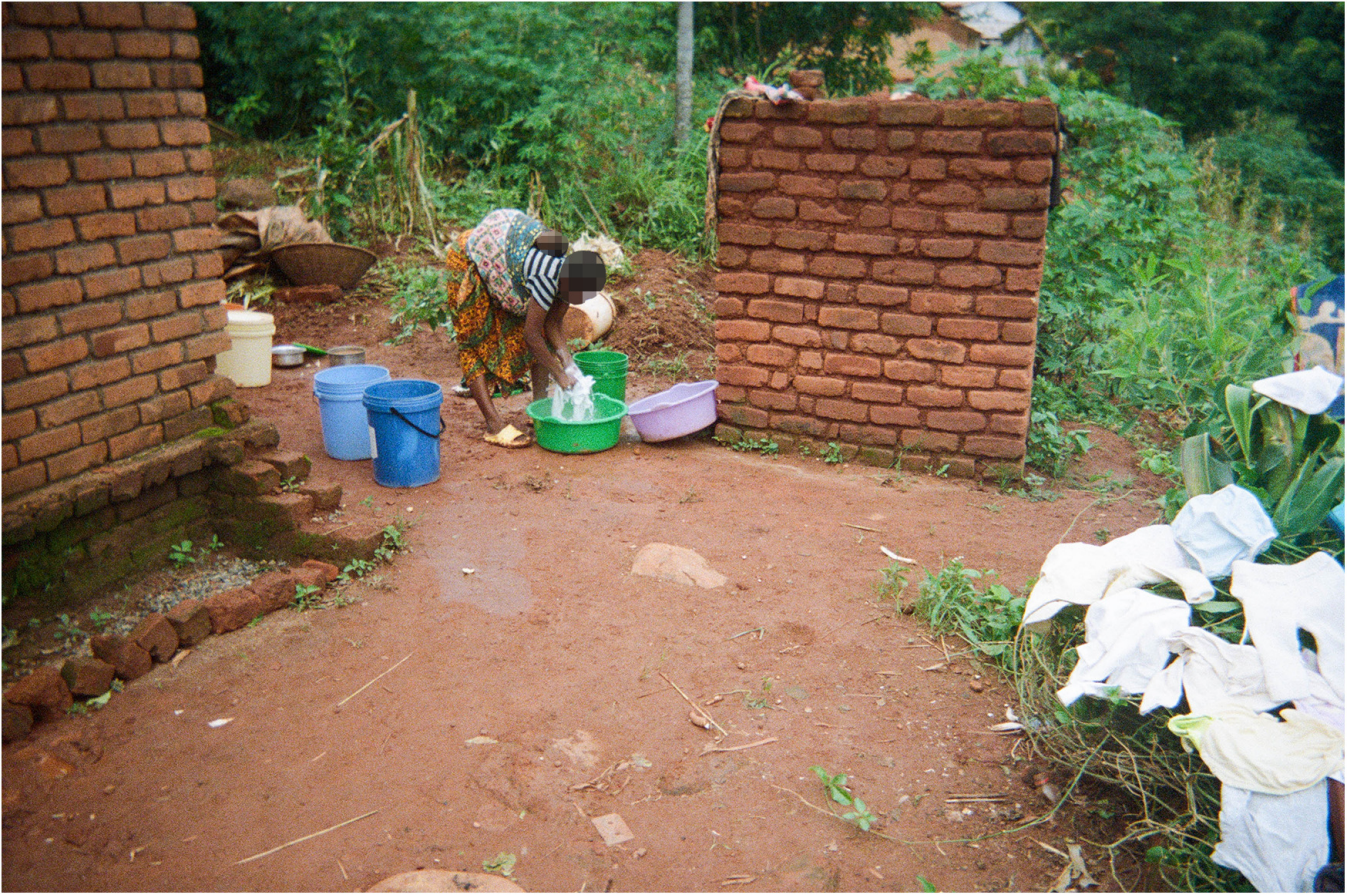
Nappies/Matewera. The photo shows a mother washing baby nappies using water she fetched from the borehole. (…) The nappies need to be changed and washed at least twice per day, but sometimes more often. Due to the insufficient water and struggle to fetch it, the nappies used during the night are accumulated until the following day, and the ones used during the day are also piled up until water is available and then all washed together, so the workload can be high. (Co-researcher 6).

Washing reusable cloth nappies, baby clothes and blankets is also an arduous task when water is scarce (see Figure 7), and mothers are forced to do this less frequently than necessary or reuse unwashed items, potentially exposing babies to additional health risks like rashes and infections.

Through their photographic evidence the co-researchers were keen to highlight that water is essential to juggle many other basic needs at once—i.e., drinking, cooking, maintaining their own personal hygiene and cleaning their homes-, as well as supporting their livelihoods—mainly crops and farming. Each of these tasks requires dedicated water, which adds to the women's burden. For example, they stated that cows are usually given a 40 liters bucket of water three times per day, so they can drink 120 liters of water per day which is a considerable amount to collect.

**Figure 8:**
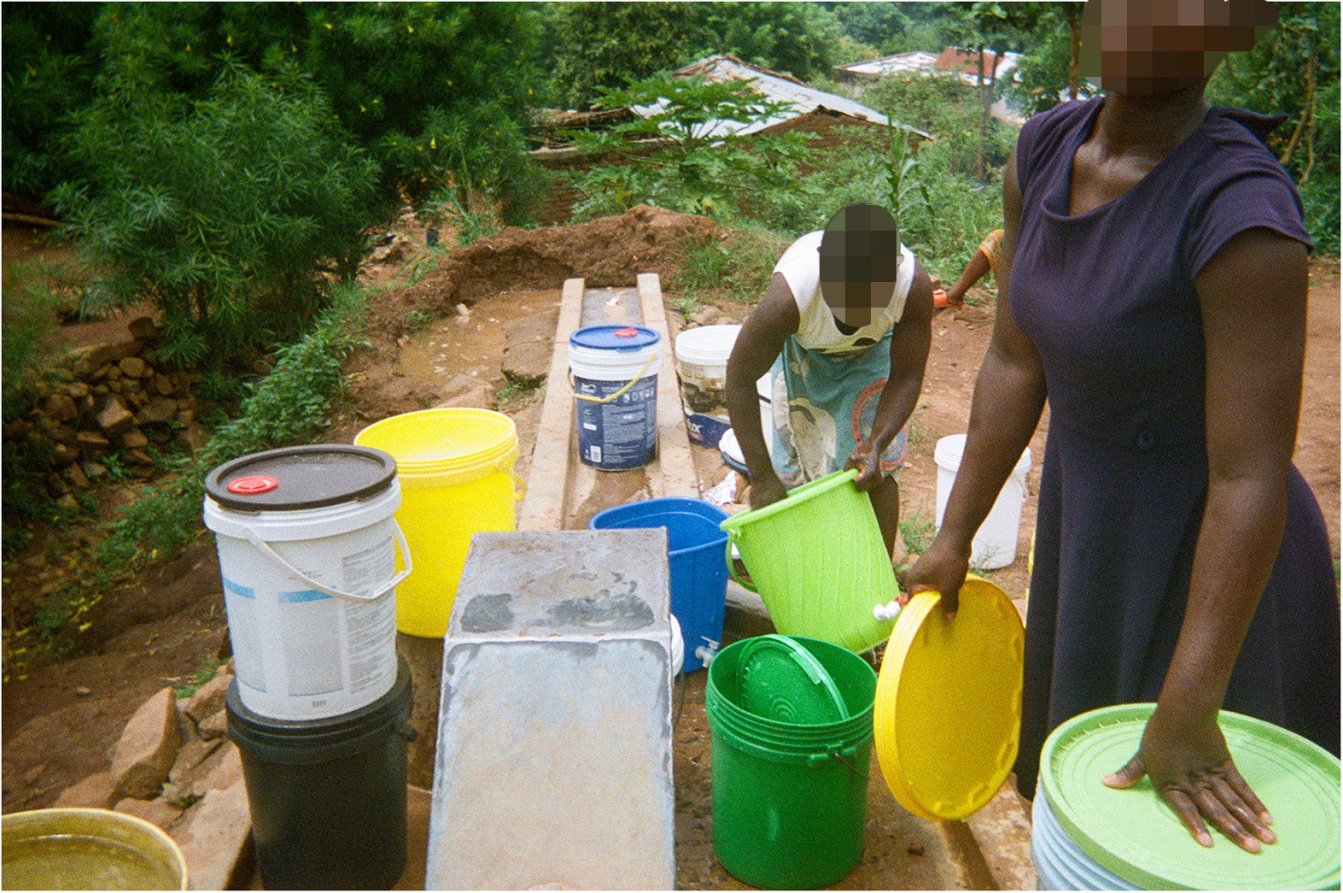
Waiting/Kuyembekezera. In this photo the ladies are fetching water for the local school. (…) I wanted to show that water is also needed for the school. I decided to title the photo “waiting” because one of the ladies is cleaning dishes and the second one is waiting her turn to access the borehole. Waiting times at the borehole are a big issue. (Co-researcher 8).

Water is also important for those collective community activities—e.g., needs of schools, health facilities, etc.—that women are expected to support, in line with local socio-cultural norms (see Figure 8).

Prioritizing water use between newborn needs and the other household and community tasks results in a precarious balance that can impact both the baby's and the family's health.

**Figure 9:**
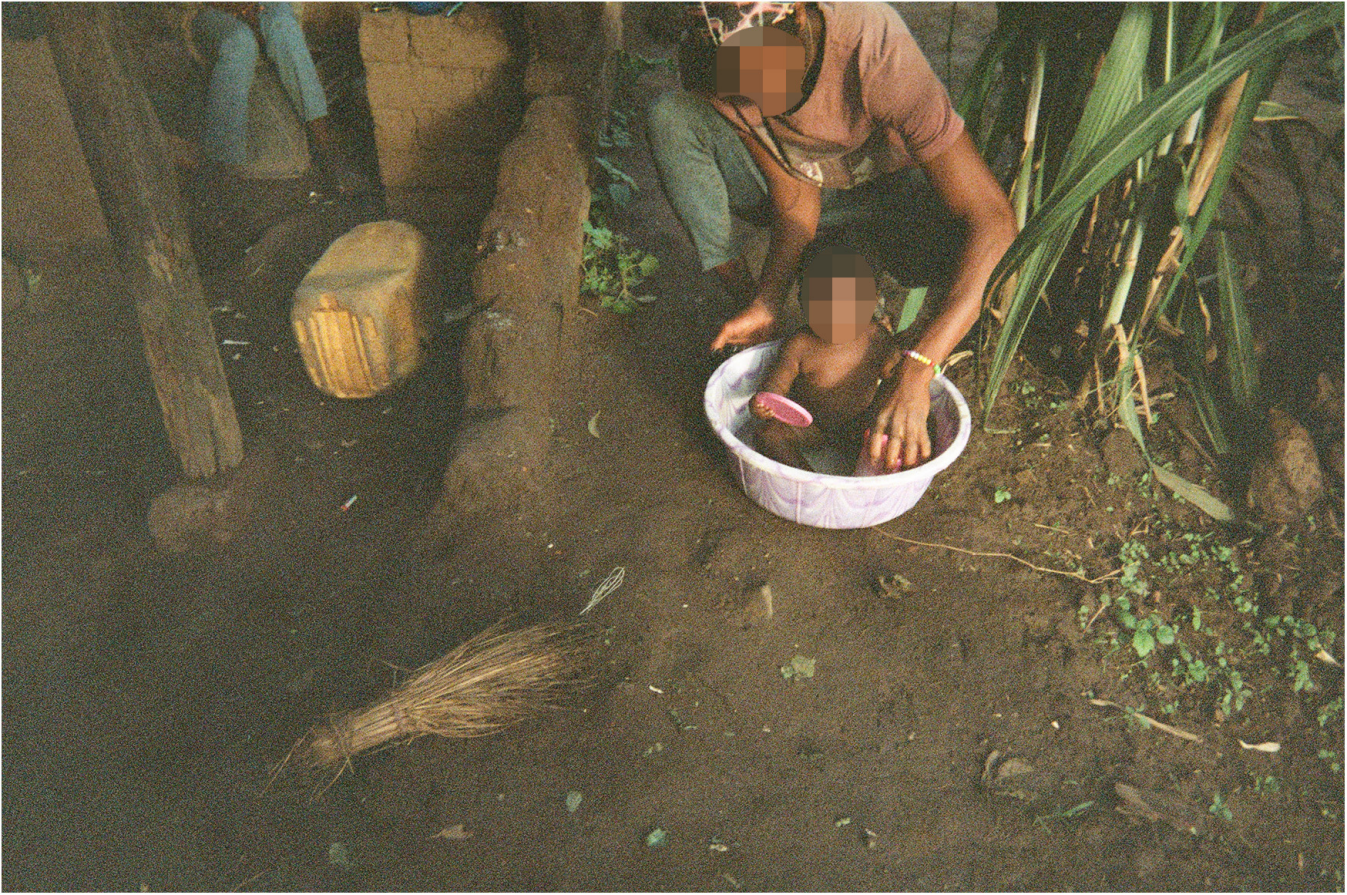
Man contribution—Abambo kutengapo mbali pa ukhondo wa mwana. In this photo a young man is bathing his baby niece (…). It is not common for a man to do house chores, but for this family the health of the baby is very important. With this photo I wanted to show that even men can help if they want, it does not have to be just the women. (Co-researcher 1).

This encourages some men to break from tradition and be more involved in the care of babies and in supporting mothers (see Figure 9). While this is not common, the co-researchers highly commended these efforts.

**Figure 10:**
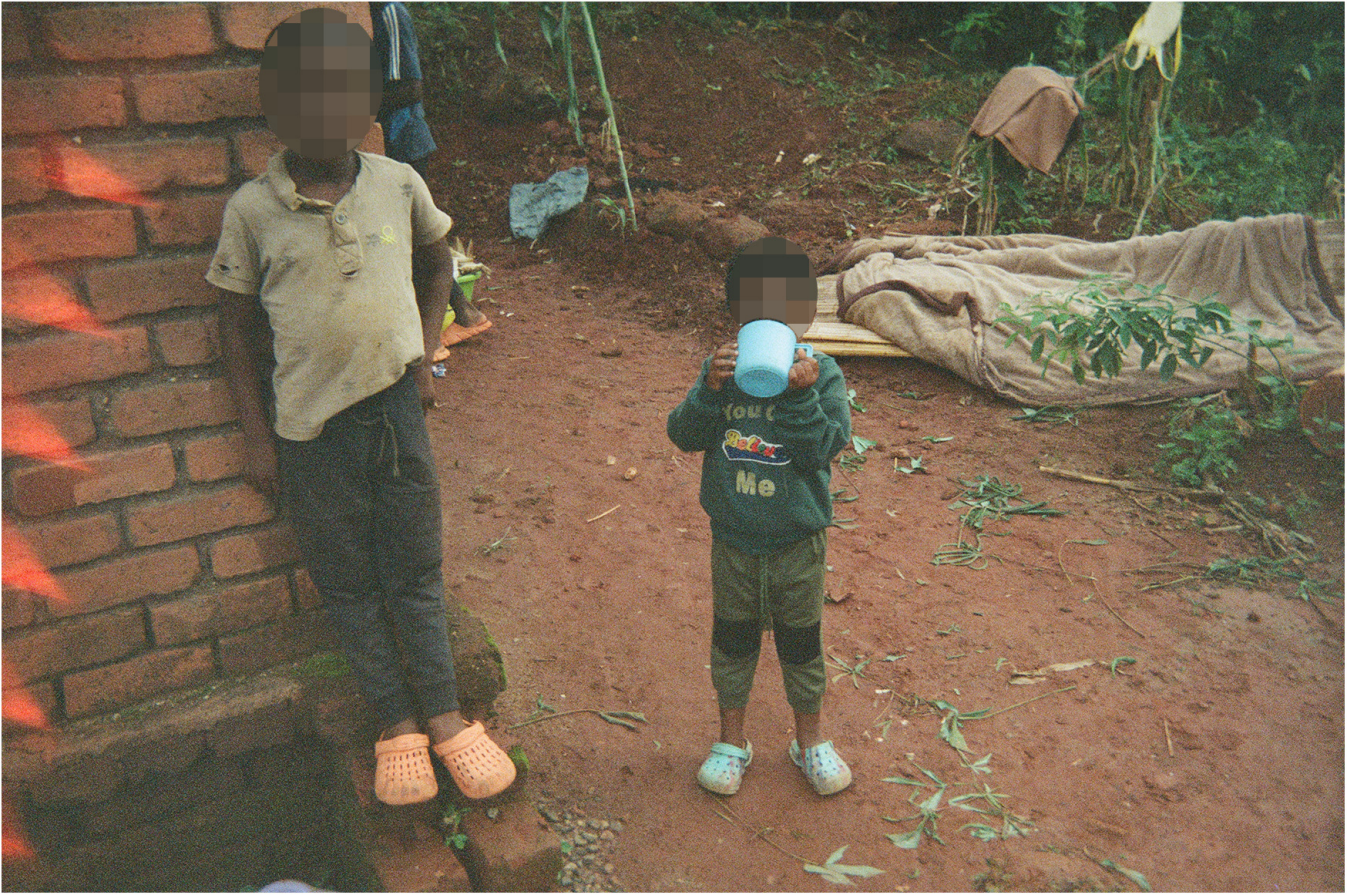
Drinking water/Madzi okumwa. This image highlights the importance of water for all age groups because it shows kids of different ages drinking water. This water (…) is good for their bodies. (Co-researcher 5).

Based on the co-researchers' narratives and photographic material it is evident that water availability or scarcity shapes nearly every aspect of their life. Using their own words, the co-researchers stated that “*water is life”,* i.e., a life-giving force seen not just as a physical necessity but the very foundation of daily survival, health and well-being, and livelihood for them, their families and communities (see Figure 10).

The centrality of water is so deeply felt that, in times of hardship, community members often join forces to improve shared water sources. For example, in this study, the co-researchers recalled how, when a water pump broke down, local residents collaborated to find a solution: “*the local people dug this makeshift well to regain access to the water. It was a huge job at the time and the whole village contributed, working together*” (Co-researcher 6).

### 3.3 Final reflections

Despite the many challenges and the modest surroundings depicted in the photographs, the narratives of the co-researchers demonstrate that maintaining cleanliness (of their babies, themselves and their households) beyond its health benefits, is also associated with a strong sense of self-respect and dignity. They were proud to show pictures of children looking “*nice and clean*”, and their homes and personal possessions being washed. This topic came up also in the discussions at the community exhibitions, demonstrating that dignity and pride are important shared values in these communities.

Yet in their final reflections, the co-researchers reiterated that the amount and quality of water available to them is just not enough to adequately take care of their newborn babies. Thinking back about their recent experiences giving birth at the local health facilities, they reported facing similar difficulties when in care. For some of them the situation back home is slightly better because they have more buckets to fetch and store water compared to when they were at the health facility; for others it is slightly worse because they have to walk longer distances to collect water. Despite these minor differences in opinions, in general all agreed that they have been struggling with water access throughout their motherhood journey and they hoped that this photographic evidence will help raise awareness about the urgent need for water solutions in their communities.

## 4 Discussion

Through the lens of photovoice, co-researchers were able to document and narrate their own stories, providing valuable insights into the lived experiences of postpartum women in rural Malawi regarding their access to and use of water.

Water access was identified as a significant challenge, increasingly compounded by the unpredictability and severity of climate change-induced weather patterns, which can affect women's health and overall recovery. Co-researchers described physical and logistical barriers to obtaining water, including long distances (especially during dry spells), unreliable supply, and the burden of carrying heavy containers—challenges that are particularly difficult for postpartum women who need rest to prevent complications like musculoskeletal disorders (40) and pelvic organ prolapse (41), among others (42, 43).

Distance, and effort to fetch water, have also been found to have a negative association with water consumption (14), i.e., mothers tend to use the water more sparingly, or rely on other less-safe water sources (8, 44). Indeed, our findings highlighted that water scarcity forces mothers to make difficult allocation choices within the household. This is an important consideration because if women do not ensure their own hydration or maintain hygiene during postpartum, this can result in dehydration, poor nutrition and increased risk of sepsis, mastitis (breast tissue infection) and other post-delivery complications (5, 42, 45, 46).

Co-researchers in our study also described how they struggled to keep newborns clean and adhere to health recommendations due to limited water availability. This can hamper newborn care in multiple ways. It impacts infant feeding—e.g., placing additional demands on mothers' time or straining their health (8, 46). It raises the risk of infectious diseases when baby hygiene cannot be maintained, and increases the likelihood of undernutrition and mortality if contaminated water is consumed (4, 6). Women in our study reported frequent cases of cholera in their communities, a problem increasingly worsened by climate change's effects on water quality and availability.

Beyond the practical challenges, the study revealed cultural expectations and norms that shape water use for postpartum women. Co-researchers documented the high demand for water within the household and the expectation that they, as women, fulfil water-related needs, even shortly after childbirth. The photographs showed that water collection is deeply embedded in cultural roles and responsibilities, which can increase the postpartum workload and psychosocial stress due to the worry of not having enough water to meet daily needs (11, 47). This insight reflects findings in the broader literature on gendered labor in low-resource settings, where water collection remains a predominantly female responsibility (48). This gendered division of labor creates additional physical and emotional burdens for postpartum women, as they are expected to perform these tasks despite the strain on their bodies during recovery.

As postpartum women in rural Malawi navigate water-related challenges, our findings show that dignity, self-respect and pride are fundamental values that shape their experiences. The contributions of family members and the wider community play a critical role in supporting or undermining these values, which in turn affects maternal and newborn health outcomes.

For example, the value of pride in motherhood is linked to the ability to care for newborns according to community standards for maternal responsibility. Women, generally, desire to be perceived as good mothers who can provide a safe, clean environment for their infants. Limited water access can threaten this pride, as mothers are unable to meet cultural expectations of newborn care, impacting both physical health and social perceptions (42).

For these reasons, family support is crucial for postpartum women. In the study, co-researchers expressed appreciation for family members who assisted with water collection or other household responsibilities. When family members help to fulfil these needs, they not only reduce the physical burden but also demonstrate respect for the woman's condition and role, which reinforces her dignity within the family. Interestingly, photos and responses from the guardians, who were also women with children, were similar to these of the mothers. In these communities, women often alternate between the roles of mothers and guardians, depending on the circumstances. This fluidity in roles may explain why there was little difference in their experiences and similar appreciation for family support.

Community members can also play a supportive role (11, 49), including through collective efforts to improve communal water sources—as described in our findings. Such efforts create a sense of solidarity and shared responsibility, highlighting the importance of social cohesion in supporting maternal health. Supportive communities enable women to feel respected and valued, highlighting the importance of social networks for maternal well-being, as they alleviate stress and enhance a woman's ability to meet health and social expectations (11).

In summary, the struggle for clean water in rural Malawi is complex. The statistical evidence in official reports provides a broad perspective on key issues affecting the water sector, but often fails to account for the complexities produced by environmental changes and spatial vulnerability (e.g., access to water sources and their shifting patterns depending on rainfall, as reported by co-researchers), socioeconomic inequality, cultural and other factors which affect the realities of communities on the ground (50). Although this study was limited by a small sample size and potential bias due to the reliance on co-researchers' personal perspectives—mitigated through a community validation exercise—photovoice still proved to be a powerful methodological tool. It enabled postpartum women, who are often marginalized in decision-making processes, to express their experiences in a compelling format. The visual nature of photovoice enriched the data by capturing environmental conditions, the physical demands of water collection, and the emotional strain of water scarcity, which may not have been as clearly conveyed through traditional research methods.

Through photographs, co-researchers were able to express not only the challenges they face but also their resilience and determination to uphold their values despite resource constraints. These experiences reflect what has been termed *everyday resilience*—the ability of individuals and communities to adapt, cope, and maintain dignity in the face of ongoing stressors and marginalization (51). In this context, postpartum women demonstrated resilience not only by navigating the physical demands of water collection but also by maintaining caregiving responsibilities and cultural expectations with limited resources. The method enabled women to share personal stories of family and community support, illustrating how these networks foster adaptive responses to chronic water insecurity. Such responses, though often invisible in policy narratives, are crucial to the women's sense of worth, pride in motherhood, and overall wellbeing. As such, photovoice provided a holistic view of their lived experiences, and allowed for a nuanced understanding of the intersection between water access, social dynamics and maternal health.

The findings from this study can be powerful advocacy tools which will be used by SURG-Water to raise awareness among decision-makers. They highlight that researching the social, economic, and cultural pathways through which water scarcity—exacerbated by climate change and environmental degradation—impacts mothers and their families is crucial for developing holistic, equitable and sustainable interventions. In doing so, it is important to adopt an intersectional approach (52) to account for how postpartum women are affected not only as women, but also as caregivers, patients recovering from childbirth, and residents of under-resourced areas—each of these roles shapes how they experience and respond to water scarcity. Recognizing these overlapping factors helps avoid one-dimensional solutions. Addressing water scarcity is rural Malawi, and other countries with similar settings, is more than an infrastructural challenge; it requires developing culturally sensitive technical interventions that account for the social contexts of water use (9), and are responsive to the specific needs of postpartum women and their communities (53).

## Data Availability

The raw data supporting the conclusions of this article will be made available by the authors, without undue reservation.
